# Epistemological and methodological paradoxes: secondary care specialists and their challenges working with adolescents with medically unexplained symptoms

**DOI:** 10.1186/s13033-018-0232-0

**Published:** 2018-09-24

**Authors:** Silje Vagli Østbye, Catharina Elisabeth Arfwedson Wang, Ida Pauline Høilo Granheim, Kjersti Elisabeth Kristensen, Mette Bech Risør

**Affiliations:** 1 Department of Psychology, Faculty of Health, UiT–Arctic University of Norway, Tromsø, Norway; 2 Department of Clinical Medicine, Faculty of Health, UiT–Arctic University of Norway, Tromsø, Norway; 3 Department of Child and Adolescent Psychiatry, University Hospital of Northern Norway (UNN), Narvik, Norway; 4 Department of Community Medicine, General Practice Research Unit, Faculty of Health, UiT–Arctic University of Norway, Tromsø, Norway

**Keywords:** Adolescents, Medically unexplained symptoms, Communication, Clinical encounters, Specialist care

## Abstract

**Background:**

Early adolescence is considered a critical period for the development of chronic and recurrent medically unexplained symptoms (MUS), and referrals and system-initiated patient trajectories often lead to an excess of examinations and hospitalizations in the cross-section between mental and somatic specialist care for this group of patients. Dimensions of the relationship and communication between clinician and patient are shown in primary care studies to be decisive for subsequent illness pathways, often creating adverse effects, but knowledge on clinical communication in specialist care is still scarce.

**Methods:**

This study explores communicative challenges specific to clinical encounters between health professionals and adolescent patients in specialist care, as presented through interviews and focus group data with highly experienced specialists working in adolescent and child services at a Norwegian university hospital.

**Results:**

The results are presented in a conceptual model describing the epistemological and methodological paradoxes inherent in the clinical uncertainty of MUS. Within these paradoxes, the professionals try to solve the dilemmas by being creative in their communication strategies; applying metaphors and other rhetorical devices to explain complex ideas; creating clinical prototypes as a way to explain symptoms and guide them in clinical action; relying on principles from patient-centered care involving empathy; and trying to balance expertise and humility.

**Conclusion:**

The challenges in communication arise as a result of opposing discourses on biomedicine, family, health and adolescence that create dilemmas in everyday clinical work. By moving away from a positivist and biomedical framework towards an interpretive paradigm, where culturally derived and historically situated interpretations are used to understand the social life-world of the patient, one can create a more humane health service in accordance with ideals of patient-centered care.

## Background

Medically unexplained symptoms (MUS) are those for which a treating physician or other healthcare providers have found no medical cause, or whose cause remains contested below [1]. Symptoms that have no definite medical diagnosis are common in all areas of primary care as well as in specialty medicine [2]. Surveys in primary care have reported prevalence rates of medically unexplained symptoms varying from 15 to 30% of consultations [3–6], and in specialist care the estimate has been as high as 52% [7]. In children and adolescents 10% to 30% report chronic somatic complaints [8–10], and these symptoms have been found to cause substantial impairment on multiple domains in everyday life, often leading to huge personal and socioeconomic costs [7, 11]. Common symptoms include fatigue, musculoskeletal pain, abdominal pain, gastrointestinal symptoms and dizziness, and typical diagnoses that are included in the category of MUS are chronic fatigue syndrome [8], fibromyalgia [12] and irritable bowel syndrome [9]. The symptoms are seen in all age groups, but early adolescence is considered a critical period for the development of chronic and recurrent somatic symptoms [13]. Most research on MUS and its inherent problems has concentrated on adults, but because symptoms often start in childhood and later develop into chronic somatization, there is growing attention to adolescents and children [14–17]. In studies on adults, it has been shown that the encounter between patients and health professionals is decisive for subsequent illness aspects, often creating adverse, somatizing effects [18–20]. Somatic symptoms and syndromes are not limited to individual bodily sensations, but are processed and developed in relational clinical contacts and health encounters, e.g. by the physician’s inclination to pursue somatic explanations and interventions [20, 21] or the patient’s need for an acknowledged diagnosis [22, 23]. Various dimensions of the relationship between health care provider and patient increase the risk of ‘dysfunctional encounters’ and iatrogenic harm. For example, studies have found that patients often have difficulties explaining the complexity of their complaints and being heard [3, 24–26], and that physicians experience difficulties in the communication and the relation with these patients and lack clear and consistent management strategies and clinical approaches [27–30]. Diagnostics have been shown to be dependent upon the medical specialty that is consulted [31], and referrals and system-initiated patient trajectories often lead to an excess of examinations and hospitalizations [32]. The health care provider’s attitudes to patients with MUS have been demonstrated to play an important role [20, 33, 34], and communication problems and challenges seem to arise when patient expectations and explanatory models of disease are incompatible [3, 24, 35]. Hence, a series of challenges and negative prospects have been shown to follow from encounters with patients with MUS in primary care, but knowledge on clinical communication in specialist care is still scarce.

### Sociocultural dimensions in the understanding of MUS

From a positivist viewpoint, diseases are considered as observable, steady and true entities, with epistemological assumptions of universal, objective facts to be identified and predicted by standardized, deductive approaches and research methods where controlled observations yield objective certainty [36]. In line with this way of thinking, the paradigm of evidence-based medicine is widely accepted as a way to support practitioners in their decision-making in order to eliminate the use of ineffective, inappropriate, too expensive and potentially dangerous practices by finding, appraising and applying scientific evidence to the management of healthcare [37].

Within a social constructionist framework, by contrast, medicine is understood as a cultural system: a system of symbolic meanings anchored in particular arrangements of social institutions and patterns of interpersonal interactions [38]. Clinical explanatory models provide a conceptual framework that allows clinician and patient to make sense of suffering and point towards possible solutions; the clinician therefore aims not only to convey objective knowledge of truth and objective certainty, but also to emotionally engage, support, motivate, change and empower the patient [39]. The language of medicine is thus not a mirror of the empirical world, but rather shaped by cultural values and different modes of knowledge, including empathetic, emotional and contextual knowledge [40]. The biopsychosocial model is one explanatory model where illness is understood as consisting of a dynamically intertwined and hierarchical system of sociocultural, mental and physiological components [41]. This model is often foregrounded as useful for the management of MUS, and is suggested as a key to *patient*-*centered care,* a framework that puts an emphasis on the therapeutic alliance, the personal experience of the patient and egalitarian doctor-patient relationships [42]. Within this framework, the aim is to reorient clinical practice around the understanding of and engagement with the patient as a person, from which follows systematic attention to the social and cultural world in which the patient lives [43]. By extension, this represents a movement away from *“one*-*person medicine”*, where the application and therapeutic techniques are a fundamentally objective issue, to *“two*-*person medicine”*, in which both the doctor’s and the patient’s subjectivity are an integral aspect of any satisfactory clinical descriptions [44]. This way of thinking has been influential in parts of specialist health care in Norway, mental health and physical medicine being typical examples.

MUS can be said to challenge the evidence-based approaches of biomedicine. In this study, our point of departure is that medical science is not only a natural science, but that it also, in its social and moral concerns, integrates elements of the human sciences [45]. Based on challenges in the intersection of evidence-based medicine and socio-cultural dimensions of clinical practice, we will explore communicative challenges specific to health encounters with adolescents with MUS, by taking a closer look at the explanatory models and rationales for clinical action that highly experienced and engaged professionals construct in order to overcome clinical uncertainty.

## Methods

### Design

The data consists of one focus group discussion with six participants, and ten individual interviews. The focus group discussion was held before the individual interviews, with the aim of familiarization with the field. The individual interviews were conducted later to obtain richer and more experience-near descriptions, and to make sure different views were represented in the data. Both individual interviews and the focus group discussion represented an interactional context for storytelling [46]. Stories are socially situated actions that are identity-giving [47], drawing on overarching cultural frameworks that include notions about ontology (what the world is made up of), epistemology (how knowledge can be acquired and verified) and morality (what is the right way to live one’s life). Drawing on strands from narrative theory, we look at the narratives constructed in the context of the interview setting as performative events, focusing on stories as collective or collaborative productions that not only take place under particular social conditions, but are social actions that construct, legitimate and maintain social realities [48].

### Recruitment and sample

We recruited highly experienced and engaged professionals to explore their experiences with and views on communicative challenges in clinical encounters with adolescents with MUS. To obtain sufficient variation of descriptions, professionals with different occupational backgrounds were purposefully selected. Participants were recruited from different departments in the adolescent and child services at a Norwegian university hospital, i.e. units specializing in child psychiatry or mental health, pediatric pain, chronic fatigue, pediatric rheumatology and adolescent medicine. Initial contact was established with leaders of the different departments, followed by several scheduled meetings to give information about the study to possible participants. Those interested in participating wrote down their names and contact information, and further arrangements were made through e-mail correspondence and by phone. All participants encountered patients with MUS in their practices, and had + 5 years (average 13 years) of experience of treatment and/or assessment of patients between the ages of 12–23. The sample consisted of three men and 13 women; six physicians, six psychologists, one nurse, two physiotherapists, and one occupational therapist. Authors IPHG and KEK conducted the individual interviews, while authors SVØ and MBR led the focus group discussion.

### Data collection

The focus group discussion lasted for 90 min and took place in a scheduled meeting at the research leader’s workplace. Prior to the meeting, the participants were given information on the study and encouraged to recall memories of particular clinical encounters with young MUS patients that they had perceived as challenging or illuminating. Constructed clinical cases were used as an elicitation technique to spark the discussion and aid the recollection of events and experiences by the participants, and a discussion guide was utilized for follow-up questions and clarifications. Questions were concentrated on thoughts and perspectives on challenges in communication, difficulties regarding cooperation between clinicians at different levels of organizations, and barriers in individual clinical encounters between practitioner and patient. Solutions and future possibilities were also discussed. An observer took notes, summarized the overall impression at the end of the interview, and sought clarity to correct potential misunderstandings.

The individual interviews lasted between 45 and 90 min, and took place at the participants’ workplaces to fit into their schedules. Interviews followed an interview guide where questions were formulated according to four research questions: (1) What is the general understanding of adolescents with MUS among health professionals working in specialist care? (2) How do they describe their experiences of working with these patients? (3) What are the main challenges that they encounter in their work? (4) How do they try to overcome these challenges?

All data were audio-recorded, anonymized and transcribed verbatim.

### Analysis

Our research team has a background from clinical psychology and medical anthropology, and had previous experience of the ambiguity and uncertainty inherent in the process of diagnostics and treatment of adolescent patients presenting with MUS, both in the capacity as researchers and as clinicians. We were therefore interested in how highly experienced professionals try to solve the dilemma of clinical uncertainty and how they describe and try to overcome communication challenges.

Initial analysis was informed by general principles for thematic analysis, following the six-phased process of coding as formulated by Braun and Clarke [49]. The analysis was influenced by both inductive and deductive reasoning, being for example based on both primary material (i.e. interview transcripts) and secondary sources (i.e. a review of the literature). The process started with intense familiarization with the transcripts, followed by initial code generation, categorization of data into tentative themes, continuous reviewing of the themes before theme definition, and finally a narrative reporting of themes across cases (see Table 1 for an illustration of the analytical process and the generation of themes, subthemes, categories and subcategories).

**Table 1:** Illustration of the analytical process with examples of themes and categories developed during analysis

Themes	Subthemes	Categories	Subcategories	Quotes
A First topic: the epistemological paradox: explaining the unexplained				
A1 Language and the dilemma of explaining the unexplained	The inherent problem in communicating inner and embodied states	Alternative approaches to communication	Using metaphors Using visual tools Connecting to the senses Play Sharing experiences	*“We have tried to close in on the problems by using visual methods. (…) For example, they have taken with them a picture they have drawn or a photo, and we have looked at it together. Or we have watched film clips that sort of captures some of the things they struggle with.”*
	The problem of diagnosis	Nomenclature Taxonomy Typification	Pragmatics Interpretation as negotiation	*“It’s not so easy to create a sense of coherence and understanding that maybe* I *have, and make it meaningful to them.”(…) “A big challenge is to create that basic mutual understanding of what this is about”*
A2 Creating explanatory models and clinical prototypes	Clinical prototypes	The “good girl”	Personality Lack of assertiveness Perfectionism Performance-oriented Pressure to achieve Workload	*“Some of them are very focused on performance and accomplishments, trying to be perfect and live up to some sort of ideal.”*
		The “trauma victim”	Negative life events Bullying Abuse Divorce Poverty Neglect	*“A lot of these patients have traumatic experiences.”*
	Explanatory models	(Bio)psychosocial model Vulnerability-stress model	Psychological factors Societal factors Acute or chronic stress	*“I’ve been wondering about the school system that we have today, that there is more pressure on young people… maybe in general in our society… that we have so much to do and are so busy. I think that I have seen an increase in tired and exhausted kids the last few years.”*
B Second topic: The methodological paradox: the uncertain expert				
B1 Empathy and the dilemma of clinical uncertainty	Focusing on emotions and relational aspects	Creating a good working alliance Building on principles from patient-centred care	Believing in the patient´s story Building trust and acceptance in clinical encounters Working towards a common goal	*“You have to believe that this is a person who really is in pain. They are really suffering. You have to believe that to create an alliance and really help them.”*
	Creating an in-group identity of engaged professionalism	Identity markers Performative aspects	Advocating patient interests Distancing themselves from biomedical parts of the health system Connecting to like-minded colleagues	*“It’s very frustrating when your referrals are declined because the person doesn’t fit the diagnostic criteria, when you know that this is a person that is suffering and could have been helped if they had been given the opportunity. That is one of our biggest frustrations. Diagnostics and the systems that we are forced into.”*
B2 The dilemma of the uncertain expert	Balancing opposing roles and tasks	Opposing roles Opposing tasks	The gate-keeper The expert The caring and nurturing helper The humble servant	*“Sometimes you feel you have some sort of diamond, that they so desperately want. And then it’s sort of up to the clinician to give an approximate evaluation of whether they should have it or not. Some of them can get very disappointed, cry or make a scene if they don’t receive the diagnosis they had expected.”*
	Relying on knowledge and experience	Tacit knowledge Years of experience Clinical intuition	Knowing their own limitations Tried techniques for themselves Having a “tool-box” of interventions and techniques Having seen several patients with similar problems Being able to seek support from experienced colleagues Being able to share responsibility with patients when appropriate	*“I’m old in the game; I have a lot of experience to keep me floated. I don’t think you should start in this job as a young psychologist or doctor. That wouldn’t work.”*

The analysis was conducted with an explorative approach, moving back and forth between the different stages. Regular meetings between the first author and the other members of the research team provided a forum to discuss and explore data collection procedures, analytical approaches, and to develop emergent ideas and interpretations.

During this process, we became interested in performative actions as well as structural elements, and explored in greater detail how the accounts were produced interactively and dialogically and hence performed narratively [46]. In this process, we also started to look for less obvious voices, hidden or taken-for-granted discourses, paradoxes, gaps and indeterminate sections that related to shared discursive practices in social, cultural and theoretical contexts [48]. By engaging in this type of re-contextualization with the research material, a larger narrative emerged about clinical uncertainty in the context of health systems trying to integrate ideals from a biomedical and positivist framework of professional certainty and evidence-based medicine with more recent ideals from patient-centered care.

## Results

In the following presentation of the results, we will provide a conceptual model of how the professionals responded to dilemmas in their everyday clinical practice, and how this translated to communicative challenges in individual encounters with the patients. The model consists of two different but connected themes: the epistemological paradox and the methodological paradox. The epistemological paradox describes two interrelated problems that both concern meaning making and interpretation: (1) finding a common language in trying to explain the unexplained, and (2) the creation of clinical prototypes and explanatory models. The methodological paradox describes the problem of combining expertise and uncertainty, and explores the devices that the professionals applied to resolve the crisis and uncertainty surrounding MUS, as represented through the two subthemes: (1) empathy and the dilemma of clinical uncertainty, and (2) the dilemma of the uncertain expert.

### The epistemological paradox: Explaining the unexplained

#### Language and the dilemma of explaining the unexplained

*“What we’re supposed to do is examine the patients, then diagnose, and then give treatment based on the diagnosis to make sure they receive the best treatment. And here you have patients that you can’t put in any category or boxes, and you don’t understand it yourself, and the patient most certainly doesn’t understand it.”*

As illustrated by the quotation above, the translation of lived experience into clusters of potentially applicable symptoms and diagnostic categories as a basis for clinical action was not a straightforward process for patients with MUS. The problem of MUS was to find a common language that could help explain and frame the puzzling symptoms. As one of the professionals explained:

*“The challenge in our work together is the language. Do we understand each other?”*

Without a shared language and understanding of the problems, the professionals’ tasks became unclear and ambiguous, creating obstacles in the clinical encounter. Creating order in the disordered by naming the problems, finding explanations and agreeing on tasks and goals was an important requisite for the patient-professional dyad to function, e.g. by creating explanatory models, guidelines and frameworks that despite ambiguities could ascribe some sort of meaning to the symptoms, and rationalize a particular course of clinical action.

Many of the professionals distanced themselves from the biomedical model of disease as an explanatory model. They perceived it as too narrow in its approach and incapable of responding to the many challenges that they were facing in their everyday clinical practice. As one of the physiotherapists explained:

*“The biomedical dualistic approach is in stark contrast to the more holistic view that my discipline is based on. I mean phenomenology… Seeing connections… Understanding the human being in its bodily expressions, as something more than just a machine that comes in with a problem.”*

The professionals distanced themselves from the biomedical metaphor of the body-as-machine, and used instead other metaphors to explain the patients’ symptoms and their work. Their work was described as *“a journey*”, “*detective work”* or *“investigative journalism”*. A psychologist described how symptoms could be traced back to difficult life experiences, the body being a container for memories, leaving marks on the body:

*“I think that burdens in life, difficult experiences, trauma, everything… The body remembers and everything is contained in the body. (…) Life experiences and the life you have led leave their marks on the body, as a pain, a stiffness, as something indefinable, as a discomfort.”*

The professionals’ understanding of symptoms was that they were metaphors for something else, the meaning of which could be uncovered in the clinical encounter. In this way, they did not see the symptoms as inexplicable, despite being medically unexplained.

To communicate their interpretations and explanations, however, was not an easy process, and several of them pointed out the limitations in the use of language for understanding and explaining the illness experience of their patients. They described how they had to be creative in the clinical encounters and in their communication strategies, for example by using visual tools like video or photographs. Many used drawings or figures to symbolize complex ideas, and others relied on metaphors as a rhetorical device.

A physiotherapist told a story about a patient with pelvic pain that she had worked with for several years; together they had created a metaphor for the patient’s body as *“a dead city”*. As their work progressed and the pain decreased, the city gradually became populated and full of life. Another professional told a story about a young boy with intense, debilitating headaches; here, they together came up with the metaphor for the symptoms as *“a wild party”.* This had enabled them to talk about what a wild party meant for the boy, and eventually his father’s alcohol problems, his difficult relationship with his father’s new girlfriend, and his parents’ divorce.

The professionals thus described being concerned with meaning making and interpretation: understanding symptoms as signs that needed to be interpreted with their patients, not as objective facts. Despite this, they also presented the process of interpretation as a negotiation process, in which they had to convince the patients to agree to their explanations so that consensus could be reached. In the focus group, two of the professionals discussed difficulties in the negotiation of meaning and understanding of symptoms:

*Professional 1:“You see it up front when you read the referrals… You know, you see at once what this is about. We sort of recognize the patients, we’ve seen it before.” (…) Professional 2: “You can sometimes anticipate that it will get difficult to create mutual understanding, it will be almost impossible to get that far.”*

In the example above, the two professionals seemed to posit the view that there existed an objective truth of causality behind their patients’ symptoms which they, based on their experience and expertise, could know up-front. In this lies the epistemological stance that one conception of reality is more real than another, and that one can uncover the objective meaning behind any given symptom independent of context. The biopsychosocial model has been criticized for precisely this paradox, i.e. that it is still caught in the separate systems view of Cartesian dualism that places different value on different types of explanations, concerning itself with finding the “right” or the “wrong” causes of patients’ suffering, and thereby excluding the patient’s illness experience [50].

#### Clinical prototypes and explanatory models

Many of the professionals claimed they were working within a holistic framework, and that they relied on the biopsychosocial model in their understanding of illness. However, despite their intentions, the analysis revealed that the professionals’ accounts mostly consisted of psychological and social explanations. They saw the symptoms as physical, but explained their causes in terms of psychological trauma, stress or personality variables, such as perfectionism or lack of assertiveness. One psychologist explained how she interpreted the symptoms:

*“I’m thinking about ‘good girls’, hard*-*working, living up to others’ expectations. I actually detest that expression ‘good girls’, but still my impression of these patients is that they’re often very concerned with achievement, want to succeed at everything, doing everything perfectly and trying to live up to some sort of ideal.”*

The quotation above illustrates how the professionals on the one hand were often wary of psychologizing patients’ problems, disliking terms like *“good girls”*, referring to the frustrations many patients experienced in the health system with assumptions of problems being *“all in their head”* when no direct physical cause could be found. Several of the professionals pointed out how mental health problems had potential stigmatizing effects, and how psychological explanations seemed to have lower status in the health system. On the other hand, they too relied on psychosocial explanations when describing clinical cases.

Two prototypical patients were presented in which the causal explanations for illness, and with them ideas of responsibility and morality, were very different. In the first prototypical category, as demonstrated in the quotation above, the patients were presented as *“good girls”,* typically excelling academically and/or in after-school activities and placing high value on personal achievement and success. The explanations for their symptoms were based on a vulnerability-stress model, one in which the patients had put too much pressure on themselves over time, failing to find ways to relax. Here the professionals’ tasks were to make the patient aware of her perfectionist tendencies, and teach her strategies for self-care. By placing the patients within an identity-bearing diagnostic category where personal characteristics were interwoven with the symptoms, the responsibility both for the symptoms development and for the treatment was transferred to the patient. By appealing to the patients’ identity as a *“good girl”,* the professional drew their attention to their moral responsibility for taking care of one’s health and making an effort to get better [cf. 45]. This placing of responsibility on the patient can be said to be in line with patient-centered care, in which the ideal is to share power and responsibility with the patients, but at the same time, it can be interpreted as serving to legitimize the professional role by lifting the burden of prognostic uncertainty.

By contrast, the other prototypical patient was presented as the *“trauma victim”.* Here the explanation for the symptoms was external factors, e.g. traumatic experiences outside of the patients’ control. The responsibility for the symptoms was placed, not on the patients, but on some unknown external factor, and typically, the family became a suspect in the explanatory model. One of the psychologists expressed it like this:

*“It’s hard to ignore the idea that their family background plays an important part. What kind of relationship they have with their parents, how much support they’ve experienced. (…)”*

There were two ways that the family could be assigned responsibility for the patients’ problems: either the primary cause as the scene in which traumatic relational events had occurred, or as a secondary cause, where the family’s responses to the symptoms or the family dynamics aggravated the adolescents’ condition. One of the physicians described her frustrations at working with families like this:

*“One period we talked a lot about pathological mothers [laughing]. Where the parents have a negative influence. They become very protective like: ‘We cannot expect her to walk outside for five minutes if she’s tired’. They contradict you when you provide some explanations, like: ‘No, we haven’t experienced that.’ They interrupt and… Yeah, kind of take over so that you aren’t able to communicate with the kid.”*

As the quotations above illustrate, in their explanatory models, the professionals presented normative ideas about the roles of the mother and father, about the nature of adolescence and relations between kin. There were several traps that the family, especially the mother, could walk into when dealing with her adolescent child: being overprotective, pushing too hard or being neglectful. Thus, the adolescent was presented as either a person that needed to develop autonomy without too much interference from the parents, or as vulnerable and in need of parental support. The mother was particularly highlighted as having responsibility for balancing and attending to these opposing needs of the child.

As this theme of the epistemological paradox has shown, the professionals work within a complex multi-layered field with several tensions and contrasting discourses on biomedicine, health, family, and adolescence. The professionals have to navigate within this field, trying to overcome communication challenges and create meaning for themselves in their work and for their patients, and attempting to create explanatory models that work as mediators to understand the symptoms and legitimize a particular course of clinical action. These explanatory models are, as we have shown, not value-free, but infused with normative ideals and morals.

### The methodological paradox: the uncertain expert

#### Empathy and the dilemma of clinical uncertainty

*“We are trained to do our examinations and to find a diagnosis, because if you don’t have a diagnosis you don’t know what to treat. And here we have a group of patients where we have to tolerate the uncertainty on the same level as them. They don’t know what’s wrong with them, and we actually don’t know either.”*

This quotation from one of the psychologists in the focus group illustrates the immense uncertainty that professionals have to endure in clinical work with medically unexplained symptoms. Professionals have to handle different levels of uncertainty: epistemic (our limited understanding of the world around us, including the lifeworld of another), ontological (our descriptions and theorizing, e.g. diagnostic categorization, can never fully capture the essence of lived experience), and prognostic (we cannot predict the future). In an attempt to deal with these many layers of uncertainty, the professionals emphasized the need to come as close to the patients’ experience as possible and believe in their suffering. These can be said to be values inherent in the concept of empathy [39]. By cultivating their empathic abilities, they could overcome some of the uncertainty of never being fully able to understand their patients’ experiences, and overcome some of the limitations they had in trying to explain and relieve their burden.

In their goal of coming close to the patients’ experiences, it also became important for them to represent another way of meeting the patient, as opposed to the typical procedure in other parts of the health system. They built their professional identity around the goal of making right the wrong that other health professionals had done by acknowledging the experiences and believing in the suffering of the patient. Many of them said that the health system was not suited to the needs of these patients, and felt the frustrations of limitations in the diagnostic language, rigid systems, and financial and bureaucratic constraints:

*“It’s very frustrating when your referrals are declined because the person doesn’t fit the diagnostic criteria, when you know that this is a person that’s suffering and could have been helped if they’d been given the opportunity. That is one of our biggest frustrations… Diagnostics and the systems we’re forced into.”*

The professionals’ descriptions suggested that they felt that their values were endangered in the current health care system, and they emphasized the need to stand together, creating a collective in-group identity of being professionals:

*“We’re the professionals, we can override decisions. And we must. (…) I think it’s our responsibility to. I mean of course we should be compliant, but not blindly so.”*

In the examples from the focus group given above, the performative role of language became evident as the participants presented themselves as professionals, deeply invested in caring for this group of patients. The pressure to handle patient interactions with great care and sensitivity was based on their knowledge that these patients often had previous experiences of referrals to numerous specialist physicians, a seemingly endless stream of diagnostic testing, the burden of medical uncertainty, and insinuations that their symptoms were only “psychological”. As one of the professionals phrased it in an interview:

*“We’re very conscious of the importance of them feeling understood and never distrusted. Because there are so many others that have distrusted them.”*

One of the most essential tasks that they faced as health professionals was thus to create an atmosphere of empathy, trust and acceptance in the clinical encounters. Their ability to do so reflected back on them as professionals, strengthening their role as capable health care providers and distinguishing them from other professionals who had failed to meet the patients’ needs. These values can be said to be in accordance with a patient-centered practice, but at the same time, they functioned to strengthen the identity and legitimacy of their professional role, thereby serving to counteract the many layers of uncertainty in the reality of their everyday clinical practice.

#### The dilemma of the uncertain expert

In dealing with patients with MUS, it was expected of the professionals that they should find a diagnosis that satisfactorily explained the symptoms of the patients and prevented further searching for answers. Going through exclusion criteria, meaning that various underlying causes needed to be checked and ruled out, was a key aspect of the diagnostic process. Many of the diagnostic labels were similar and had overlaps, but only some legitimized the patient’s sick role, providing access to publicly funded treatments or social benefits. Thus, the professionals took on the role of a gatekeeper, deciding who deserved to enter the sick role. How patients’ symptoms were explained was thus of great importance in the diagnostic process, involving different ideas of morality and responsibility. The systems within which the professionals worked were often seen as the end of the road for the patients, and an important task for the professionals was therefore to reassure them that no more examinations and testing were needed, putting a stop to further referrals and system-initiated patient trajectories. One of the physicians described the diagnostic process as follows:

*“Sometimes you feel you have some sort of diamond, that they so desperately want. And then it’s sort of up to the clinician to give an approximate evaluation of whether they should have it or not. Some of them can get very disappointed, cry or make a scene if they don’t receive the diagnosis they had expected.”*

In this description, the professional role is that of an expert or gatekeeper with a firm grip on the answer, the diagnosis, or the *“diamond”*, legitimizing the symptoms for some patients and not for others. In this role, the professionals presented an attitude of suspicion and distrust, aiming at exposing malingerers. Implicit in this lay the biomedical assumptions that they as experts could provide value-free certainty and context-independent truth. This role stood in stark contrast to the caregiving role they had in the context of treatment, where they described the importance of believing in patients’ suffering, listening empathetically to their story and supporting them in their process towards recovery.

Many of the professionals emphasized that an important part of being able to perform their job was that they had several years of experience. It was of value to have experience that could aid them in their interpretations and give a sense of certainty. The years of experience gave them a form of tacit knowledge and clinical intuition, providing them with the necessary tools for being able to stand firm in difficult situations and balancing the different roles they had in relation to the patients. It also gave them legitimacy when talking to their patients, helping them in their work of reassurance and trust building.

*“Clinical experience and clinical intuition, that’s really important for being able to handle this job. It actually helps to have a few grey hairs. They [the patients] can tell that I’ve been around the block, so they can’t just… I look at them and ask ‘Do you think I’ve seen this before?’ and they say to me ‘Yeah, I bet you have.’”*

As the above quotation from a physician shows, clinical experience not only built a sense of certainty in an uncertain and ambiguous field, but it also created legitimacy for them in their professional role, as someone who could be trusted and whose opinions were of value and should be respected. Despite this, many of the professionals said that they did not conceive of themselves as experts, pointing out that it is the patients that do the work in the healing process, by listening to their own bodies and making changes in their lives. As one of the physicians said in an interview:

*“I always say, you’re the expert, I’m only the doctor. I have to learn from you.”*

This sharing of power and responsibility can be said to be in line with patient-centered care. However, the previous examples also show the contrasting roles the physicians assumed, sometimes placing emphasis on themselves as *“professionals”* with knowledge and experience that could provide them with certainty and guide them in their attempts to give advice or present solutions, while at other times they presented themselves as humble servants without clear answers and merely supporters of the patients’ own processes.

## Discussion

We have presented a conceptual model for communication challenges in the context of clinical uncertainty consisting of two interrelated paradoxes: the epistemological paradox of explaining the unexplained, and the methodological paradox of the uncertain expert. We have demonstrated the many dilemmas inherent in the uncertainty of MUS that professionals face in their everyday clinical practice, and have shown how they try to solve these dilemmas and navigate within the many complex and disparate discourses on biomedicine, health, adolescence and family.

The epistemological paradox concerns the problem of meaning making and interpretation, and the translation process of experiences and phenomena in the world into concepts that we can understand. Our language not only represents the world, but also creates the world through the interpersonal process of interpretation and meaning making [47]. The problem of MUS can be said to result from the difficulties in conceptualizing and framing symptoms within the theoretical models and taxonomies represented by the biomedical framework [3], and the translation of complex theoretical ideas into the understanding of individual cases [51].

The explanatory models created by the professionals provided them with a conceptual framework that allowed clinician and patient to make sense of the puzzling and disturbing phenomena that MUS represents, making the suffering tolerable by creating meaning and pointing towards possible solutions. As such, the explanatory models not only aimed at conveying objective knowledge of truth and certainty, but were also created to emotionally engage, support, motivate, change and empower the patient [43].

The professionals claimed to be working in accordance with a biopsychosocial model. However, the professionals’ accounts clearly revealed that to work within this framework may have been an impossible ideal to live up to in the everyday reality of their practice [cf. 30]. Instead, the explanatory models that the professionals used relied on clinical prototypes that were based on the knowledge provided by their many years of experience, describing how illness could result either from personality traits or from dimensions within the family [cf. 45]. The prototypes served as mental shortcuts that could guide them in their everyday practice, lifting the burden of medical uncertainty. In this way, their approach can be said to be pragmatic, trying to capture both the unique in each patient’s story, but at the same time giving them a general understanding that could be applied in their clinical decisions. Studies from general practice have also demonstrated that the epistemological incongruence between disease models and the reality of clinical practice is managed in a more flexible and pragmatic way with more experience [52].

However, as we have shown, the professionals’ explanatory models were not value-free, but were infused with normative and moral imperatives on what constitutes a good life, a good family and a good adolescence. These moral imperatives shifted the responsibility for the symptoms and for the treatment over to the patients in some cases, and to the family (and especially the mother) in others. This tendency to place responsibility on the patients and their families has also been pointed out in previous research, and the concept of “blaming the mother” has been amply demonstrated in studies on family welfare, adolescent health and child protection work [45, 53, 54].

The professionals tried to overcome the challenges of interpretation and meaning making by relying on alternative forms of communication. They showed a high level of creativity in their application of rhetorical devices and visual tools, like the use of metaphors or reliance on photos or video. The reliance on metaphors to explain symptoms and break down complex ideas into something that can be grasped on a more concrete level has also been demonstrated in other studies as a valuable strategy for handling uncertainty and overcoming communication challenges in clinical encounters [55], especially relevant to adolescents with MUS [56].

The professional ideals of our participants were grounded in a phenomenological and interpretive framework, placing value on subjectivity and trying to capture the patient’s own experience of his/her lifeworld. However, at the same time the participants emphasized their professional expertise and abilities in revealing the objective truth behind the presented symptoms. In this way, the professionals’ accounts demonstrated the multilayered and complex nature of meaning making in clinical work, in that they seemed to create narrative threads from competing paradigms and knowledge regimes at the same time. Studies from general practice have proposed that the problem with MUS for physicians is the epistemological incongruence between learnt ideal disease models, and the reality of meeting patients suffering from persistent illness and distress [52]. This incongruence also seems to exist in specialist health systems, perhaps as a result of the different paradigms and epistemological realities that frame the health system [39]. Much of the somatic health system is founded on a biomedical positivist paradigm where clinicians are seen as experts who should find the cure for the diseased part of the body-machine and replace it. In psychotherapy and mental health domains, however, the ideal is an interpretivist paradigm where patients’ symptoms are understood as signs to be interpreted and where healing is a complex interpersonal process of meaning making. Consequently, adolescent patients with MUS are confronted with a health system that is divided in its understanding of their illness.

The methodological paradox concerns the fact that the theoretical underpinnings for understanding the most suitable methods, or best practices, for specific cases, were not compatible in a coherent methodology. This is illustrated by the many opposing and incompatible tasks that the professionals were expected to perform, and the opposing and conflicting roles in the clinical encounters. The professionals tried to overcome the challenges in combining the role of the expert and the uncertainty inherent in the phenomenon of MUS by relying on empathy in the clinical encounters, acknowledging their patients’ suffering and aspiring to make the patient an expert on his/her own illness experience. The importance of relational factors like trust, empathy and emotional support in clinical encounters with MUS patients has also been demonstrated in studies from general practice [34, 52, 55]. These ideals can be said to be in accordance with patient-centered practice, and at the same time, they functioned to strengthen professional identity and lift the burden of prognostic uncertainty.

Despite the challenges they experienced and the many paradoxes and dilemmas they were confronted with in their practice, the professionals all claimed that they enjoyed working with this group of patients, presenting themselves as high in expertise and having the necessary capacities to do their job in a satisfactory way. This finding stands in contrast to research from general practice, where the overall picture is that physicians find encounters with MUS patients strenuous and troublesome, the patients often being described as difficult and demanding [18, 27, 29, 30]. The professionals in our study also described challenges in their work, but at the same time they felt that their work was meaningful and fulfilling. Their accounts demonstrated that they were highly invested, building their professional identity around their ability to help and support their patients and offering them something that other health professionals had failed to provide. Being the last resource in a long line of medical encounters for the patients, and also having made a deliberate choice of this line of work through their specializations in pediatric medicine or mental health care, they perhaps felt greater pressure and demands than GPs for finding strategies and solutions to solve the clinical dilemmas and cope with the uncertainty. As we have shown, the professionals assumed a pragmatic and creative attitude in handling their demanding work, both in their communication strategies, their explanations and creation of clinical prototypes, and in their use of empathy and the balancing of expertise and humility in relation to their patients.

### Strengths and limitations

Most studies on communication challenges in clinical encounters are from primary care with adult patients [see 52]. Adolescents are a patient group which in terms of health behavior is ‘in the making’, where lifelong patterns of self-management of and adjustment to chronic health conditions are established [57]. Research contributing to the understanding of specific challenges in clinical work with adolescents presenting with MUS is therefore of great value.

A limitation of the study is that the findings reveal the professionals’ perceptions and interpretations, and do not necessarily reflect what is actually happening in encounters with patients. Such issues should be the subject of observational studies. In the literature on MUS, there is a clear gender difference [2, 5, 58]. It is a limitation of our study that this issue was not included in the interview guide and research questions. To investigate gendered issues should be a topic in future research. Moreover, norms and conventions may influence interview responses and there might have been a certain discrepancy between what the professionals actually thought and what they said. Further, the relatively small sample of professionals from different areas in the health system makes it difficult to draw general conclusions. Accordingly, the findings should not be regarded as a reproduction of reality, but rather a reflection and an interpretation of a reality described by these professionals at a given time and place.

## Conclusion

The study illustrates the many dilemmas that professionals working with adolescents with MUS face in clinical encounters, and shows how they try to solve these dilemmas pragmatically to meet their patients’ needs. The use of alternative and creative methods of communication seems especially productive for overcoming communicative challenges in clinical encounters with adolescent patients with MUS, and should be studied further. The study also demonstrates the limitations of the biomedical systems of classification on which the paradigm of evidence-based medicine is based, when managing patients with MUS. The idea of medicine being context-independent and able to provide value-free certainty, even with well-known somatic diagnoses, can be said to be an illusion presented by “the voice of medicine” that creates difficulties and communication challenges in clinical encounters and across health systems [59, 60]. The application of the generalized truths of biomedical science to the unique context of an individual patient’s life and circumstances will always be uncertain [51]. By moving away from a positivist and biomedical framework towards an interpretive paradigm, where culturally derived and historically situated interpretations are used to understand the social life-world of the patient, placing value on subjectivity, reflexivity and contextuality in the process of clinical understanding, one can create a more humane health service in accordance with ideals of patient-centered care [43].
